# Real-Time Walk Error Compensation Method Using Echo Signal Magnitude Measurement in ToF Laser Scanners

**DOI:** 10.3390/s24030733

**Published:** 2024-01-23

**Authors:** Bartosz Sędek, Marek Zygmunt, Marcin Jakubaszek, Tadeusz Drozd, Jacek Wojtanowski

**Affiliations:** Institute of Optoelectronics, Military University of Technology, 2 Gen. S. Kaliskiego Street, 00-908 Warsaw, Poland; marek.zygmunt@wat.edu.pl (M.Z.); marcin.jakubaszek@wat.edu.pl (M.J.); tadeusz.drozd@wat.edu.pl (T.D.); jacek.wojtanowski@wat.edu.pl (J.W.)

**Keywords:** walk error compensation, echo signal magnitude measurement, laser scanner, time-of-flight measurement, laser pulse energy

## Abstract

The rapid advancement of mobile laser scanner technology used for terrain mapping, among other things, imposes increasing requirements for scanning frequency and distance measurement accuracy. To meet these requirements, rangefinder modules are expected to operate with high echo signal dynamics and to allow accurate distance measurement even based on single-laser-pulse echo detection. Such performance can be potentially achieved using pulsed time-of-flight (ToF) laser rangefinders (LRF). In conventional ToF modules, however, the STOP signal (for time counter interruption) is generated using a straightforward fixed-threshold comparator method. Unfortunately, it corresponds to the so-called walk error, i.e., the dependence of the measured time of flight on the magnitude of the echo signal. In most ranging applications, however, the LRF detection channel can be exposed to an extremely large span of received echo power levels, which depend on the distance measured, type of target surface, atmospheric transmission, etc. Thus, the walk error is an inseparable element of the conventional ToF technique and creates a fundamental limit for its precision. This article presents a novel method of walk error compensation in real time. By using our authorial electronic circuit for measuring the magnitude of the echo signal, it is possible to effectively compensate for the walk error even when the echo signal brings the detection channel amplifiers into saturation. In addition, the paper presents a laboratory method for calibrating the walk error compensation curve.

## 1. Introduction

**Figure 1:**
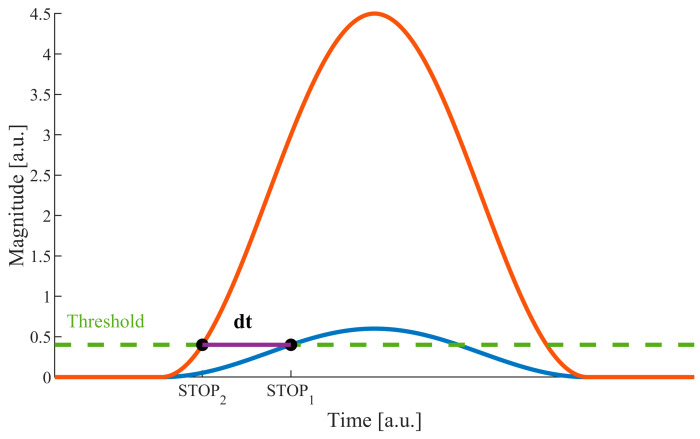
Walk error at different signal levels (*dt*—timing error depending on the magnitude of the echo signal).

In time-of-flight (ToF) laser rangefinders, distance is determined by measuring the time it takes light to travel to the target and back. Thus, technically, this method involves measuring the time period between START and STOP signals. The first signal is emitted synchronously with the emission of the optical pulse and starts the timer. The second signal is generated by the received optical echo signal and causes the counters to stop. It can easily be noticed that if the STOP signal is generated through a fixed-threshold comparator, the magnitude of the echo signal strongly affects the moment of time at which the STOP is generated (Figure 1).

This influence is called walk error and is the source of the largest errors in the ToF method of distance measurement. In order to analyze this effect quantitatively, we developed the following mathematical approach. The power of the optical echo signal received by the detector (*P_s_*) can be described by Equation (1):

(1) $$P_{S}(R)=OL(R)*\frac{P_{T}\rho D_{r}^{2}\eta_{r}\eta_{f}e^{-2R\gamma }}{4R^{2}}$$

where *OL*(*R*) is the overlap factor depending on the distance and design parameters of the rangefinder, *P_T_* is the transmitter power, *ρ* is the reflection coefficient (reflectance), *D_r_* is the diameter of the receiving lens, *η_r_* is the transmission of the receiving lens, *η_f_* is the transmission of the optical filter, *γ* is the atmospheric extinction coefficient, and *R* is the distance to the object.

**Figure 2:**
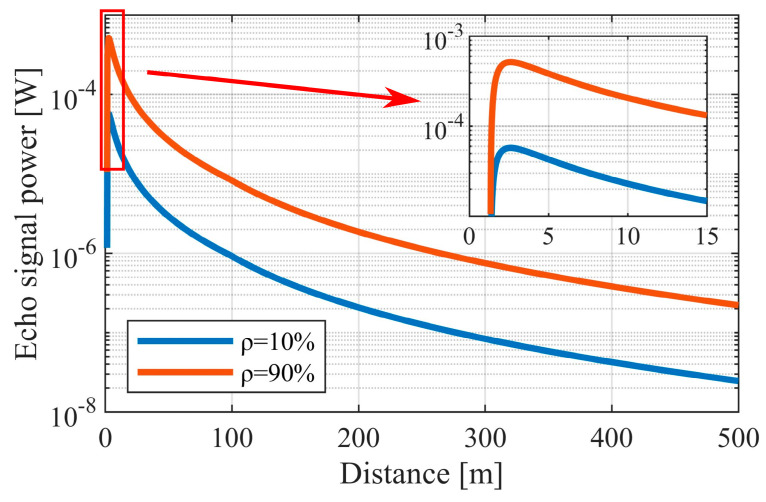
Examples of echo signal power range dependence for two reflection coefficients of the target. The area with a strong influence of the overlap factor (red rectangle).

Based on Equation (1), a pair of sample curves showing optical echo power range dependence for two values of reflection coefficient were plotted (Figure 2).

Based on the obtained results, it can be concluded that even for small distances, a change of the echo signal of several orders of magnitude can be expected. Therefore, it is evident that the discussed walk error cannot be omitted. In order to minimize its impact on ToF precision, it is necessary to use a method to reduce the effect of signal magnitude on the STOP signal’s timing.

Methods that reduce or compensate for this error can be divided into digital and analog. In analog methods, the walk error is compensated for by transforming the echo signal pulse from unipolar to bipolar [1], by knowing the duration of the echo signal [2,3]; through automatic gain control, which stabilizes the magnitude of the echo signal [4,5,6]; by measuring the duration of the pulse and the rise time of its edge [7,8,9,10]; through CWT (continuous wavelet transform) [11]; or by using a delay line [12]. Digital methods that determine the STOP signal can operate on a digitally recorded echo signal [13,14,15,16]. They also make it possible to distinguish overlapping echo signals [15,17], which is not possible in analog methods. Digital methods allow reconstructions of signals that bring detection channel amplifiers into saturation [18]. Another way of dealing with the discussed error is to suppress the echo signal dynamics using optical methods [19]; however, this requires the application of sophisticated freeform components.

This paper presents an analog walk error compensation method based on echo signal magnitude measurement. The measurement of echo signal magnitude was implemented using the ESMMC (Echo Signal Magnitude Measurement Circuit) presented by the authors in the [20]. In addition, the article presents a new laboratory method for calibrating the walk error compensation curve.

## 2. Walk Error Analysis

The effect of the echo signal magnitude on the timing error, which depends on the signal parameters and the detection threshold, can be analyzed using the sin^2^-shaped pulse as an example. Described in this way, the echo signal (unlike a Gaussian curve) has a finite duration and therefore a precisely defined onset. This is essential when analyzing signals with high dynamics. Such a pulse shape is described by Equation (2):

(2) $$U\left(t\right)=\left\{\begin{array}{c} A{\left[\sin\left(\frac{t*\pi }{2*\tau }\right)\right]}^{2}\;\;for\;t\in <0,\;2\tau >\\ 0\;\;\;\;\;\;\;\;\;\;for\;t>2\tau \end{array}\right.$$

where *A* is the peak value; *τ* is pulse time width at the full width half maximum (FWHM).

In the fixed-threshold method, the time moment, *t*, for which the value of the echo signal is equal to the value of the comparative threshold (*U_th_*) is looked for. In order to determine the time of generation of the STOP signal in the discussed example, Equation (2) was solved against the assumed comparative threshold, *U_th_*. The generation time of the STOP signal as a function of the threshold value is described by Equation (3):

(3) $$t=\frac{2*\tau }{\pi }*asin\;(\sqrt{\frac{U_{th}}{A}})$$

Assuming that a laser rangefinder emits pulses of a stable shape and duration, the walk error can be determined as the difference, dt, between the moments of *STOP*_1_ and *STOP*_2_ signal generation, for two signals of different magnitudes (*A*_1_ and *A*_2_). Considering an echo signal of magnitude *A*_1_ and another echo signal (*A*_2_) that is k times larger than *A*_1_, the error, dt, can expressed by Equation (4):

(4) $$dt=\frac{2*\tau }{\pi }*[\mathrm{asin}\left(\sqrt{\frac{U_{th}}{{k*A}_{1}}}\right)-\mathrm{asin}\left(\sqrt{\frac{U_{th}}{A_{1}}}\right)]$$

**Figure 3:**
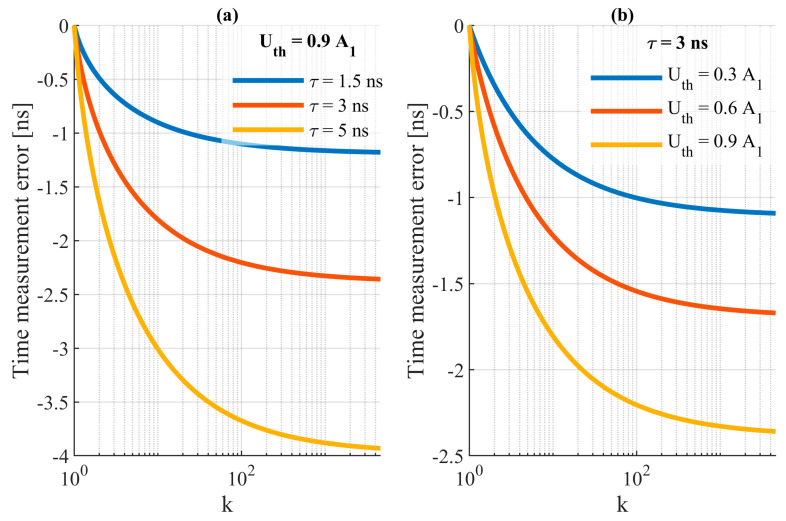
STOP timing error characteristics (**a**) for a fixed comparative threshold, *U_th_* = 0.9*A*_1_, and (**b**) for a echo signal duration of *τ* = 3 ns.

Equation (4) shows that the timing error depends on the signal duration (*τ*), the comparative threshold (*U_th_*) and the ratio of the magnitude of the *A*_1_ and *A*_2_ signals. Figure 3 shows the characteristics of the STOP timing error as a function of these parameters.

Figure 3a shows the effect of the echo signal pulse duration with the comparison threshold set at 90% of the *A*_1_ signal magnitude. It is evident that the echo signal duration has a strong effect on the timing error, which linearly depends on *τ* (4). Figure 3b shows the effect of the comparative threshold level changes for a signal with a fixed duration of *τ* = 3 ns. The characteristics show that the walk error can be reduced by shortening the signal duration or by lowering the value of the comparative threshold.

In order to obtain shorter echo pulses, one would have to increase the bandwidth of the receiver and also face the difficulty of generating short enough strong optical pulses.

On the other hand, when lowering the comparative threshold, the increased probability of a false alarm must be taken into account. Assuming that the noise has a normal distribution, the probability of a false alarm for a threshold of 3*σ* is about 1.3 × 10^−3^, and that for a threshold value of 6*σ* is about 1 × 10^−9^, where the physical meaning of σ is the effective voltage of the noise.

This shows that both methods mentioned above are not optimal. For this reason, we proposed another approach [20]. It was proven that in order to effectively compensate for the walk error, it is possible to use the knowledge about the absolute magnitude of the echo signal. Based on this value, an appropriate correction can be made to the timing result. The main challenge, however, is to be able to measure this magnitude even if the circuit is saturated. Equation (1) shows that the dynamic range of the echo signal can be at least several thousand, which means that the effects of saturation are inevitable. For this reason, we proposed the novel electronics solution called the Echo Signal Magnitude Measurement Circuit (ESMMC), which allows signal magnitude measurement even if saturated [20].

## 3. Measurement Circuit Calibration Method for the Walk Error Compensation Algorithm

**Figure 4:**
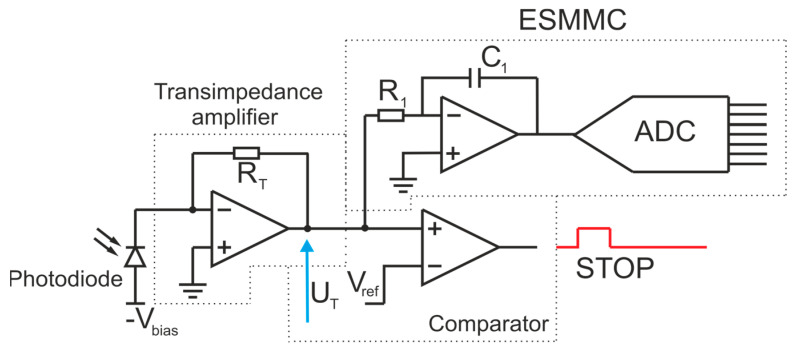
Block diagram of the measurement system [20].

To measure the magnitude of the echo signal in the tested rangefinder, the ESMMC module was used. Its operation is based on the integration of the voltage signal from the output of a transimpedance amplifier. A simplified schematic of the receiver is shown in Figure 4.

**Figure 5:**
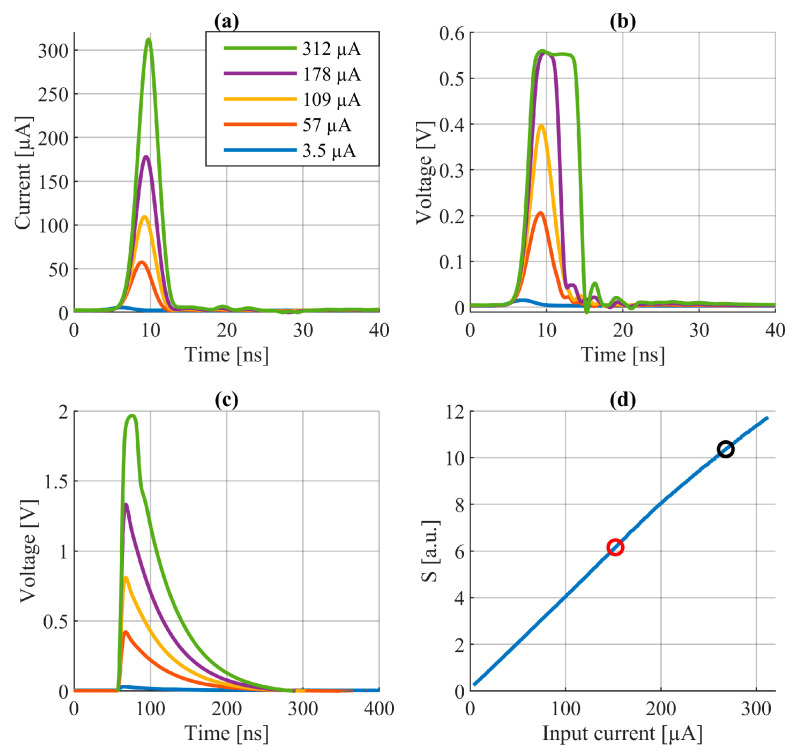
Test results of the ESMMC (**a**) echo signal at the input of the transimpedance amplifier, (**b**) signal at the output of the transimpedance amplifier, (**c**) signal at the output of the integration circuit, and (**d**) conversion of the magnitude of the echo signal “a”, into the area under the integration curve “c” [20].

The use of the ESMMC circuit, unlike, for example, a peak detector, allows the measurement of the magnitude of the signal even when it is saturated. This is illustrated in Figure 5d, where it can be seen that the area under the signal at the output of the integration circuit (S) is proportional to the photocurrent of the diode, even when the transimpedance amplifier (red point) and the integration circuit (black point) are saturated.

The measurement of saturated signals is important from the point of view of distance measurement correction, because, as was shown in Section 2, even in this range the effect of a change in magnitude on the measurement can reach several centimeters. An additional advantage of the ESMMC is that it returns the result in real time, and more importantly before the next laser pulse is emitted. This makes it possible to correct distance measurement results directly in the rangefinder, in real time too.

In order to apply the discussed solution of walk error compensation in a real rangefinder, it is necessary to carry out the calibration of its detection channel. This has to be performed to obtain the dependence of the distance measurement error on the echo signal’s magnitude. Such a walk error compensation curve has to be determined for each rangefinder individually. This is due to the natural differences caused by the accuracy of electronic components and differences in the shape of the generated optical signals. The calibration process can be implemented by measuring the distances and magnitudes of the echo signal from objects with different reflection coefficients located at precisely determined distances from the rangefinder. However, such a method of calibration is extremely time-consuming and difficult to implement due to the need to precisely position reference objects at multiple distances in terrain. Thus, such an approach seems to be completely ineffective if mass production is taken into account.

**Figure 6:**
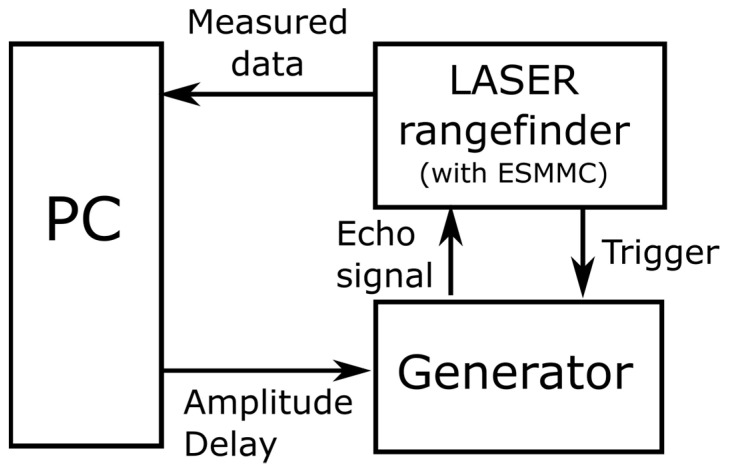
Schematic of the test stand (PC—computer).

In order to facilitate this calibration process, we propose a new method that can be realized on a compact test bench within a lab room. The idea of this method is shown in Figure 6. The calibrated laser rangefinder (LRF) receiving electronics with the ESMMC implemented receives electrical signals from the generator. The pulses from the generator have an adjustable maximum value (simulating the change in the power level of optical echoes) and an adjustable delay time relative to the start (simulating the distance). All parameters of the pulse from the generator, as well as the data read from the rangefinder, are recorded by an application on a PC.

The presented scheme allowed us to experimentally determine the impact of the echo magnitude on the distance measurement error. For this purpose, first of all, the magnitude of the generated pulses (provided to the LRF) were changed for each selected delay. Then, a similar procedure was performed in a reversed manner—for each set magnitude, a series of measurements with a changing delay was recorded. The mean values and standard deviations of the measured distance and signal magnitude were determined for each series. The test was repeated for several different delays to verify the performance of the method over the entire rangefinder measurement range.

**Figure 7:**
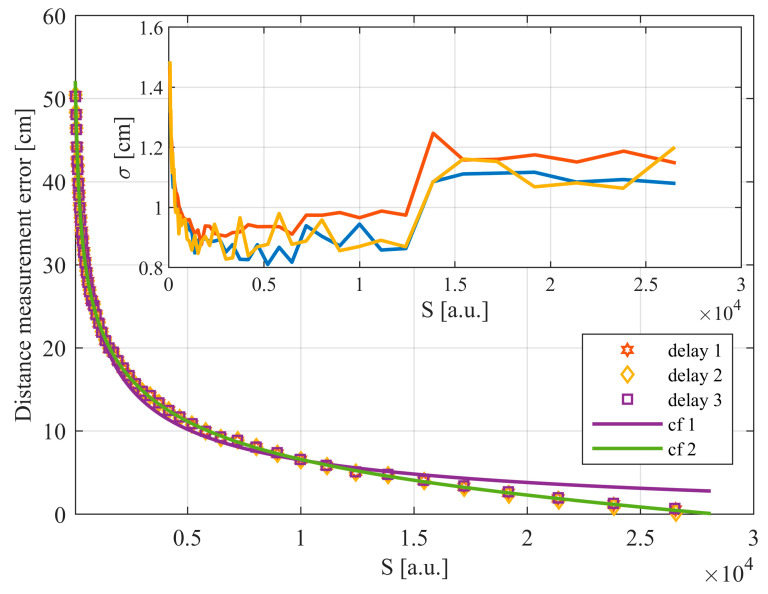
The mean error and standard deviation of the measured distance as a function of the echo signal magnitude (S).

Based on the average values determined during these tests, distance measurement errors were calculated as the difference between the distance resulting from the delay and the average measured distance. This made it possible to obtain the dependence of the distance measurement error as a function of the echo signal magnitude. The family of plots, showing this dependence for three different delays, is shown in Figure 7.

Based on the characteristics shown in Figure 7, a compensating curve matching the results can be determined (“*cf 2*” curve). This makes it possible to improve the accuracy of distance measurements to less than ±0.5 cm. During the tests, it was noted that the echo signal saturated the first amplification stage at the measured value corresponding to *S* ≈ 2900. This confirms the importance of using the ESMMC, since in the saturation range, without the ESMMC, the error would have exceeded 14 cm.

Figure 7 also shows the curve obtained from Equation (4) (“*cf 1*” curve). This allows us to partially compensate for distance measurement errors, but has deviations from the results, especially for large signals. Deviations of the actual results from the model are caused, among other things, by differences in the shape of the echo pulse, the limited bandwidth of the receiver, and signal distortion associated with the nonlinear operation of amplifiers. Due to these phenomena, each rangefinder requires the development of a dedicated walk error compensation curve.

Two laser scanner rangefinder modules were calibrated using the presented method. The first scanner is based on a semiconductor laser (SLS—semiconductor laser scanner) working at 905 nm wavelength and offering the maximum range (to the target *ρ* = 11%) of ~110 m. The second one is equipped with a fiber laser (FLS—fiber laser scanner) operating at 1550 nm wavelength. The maximum range of the FLS (to target *ρ* = 14%) is ~300 m. After calibration in accordance with the described methodology, the scanners were subjected to field tests.

## 4. Field Tests

**Figure 8:**
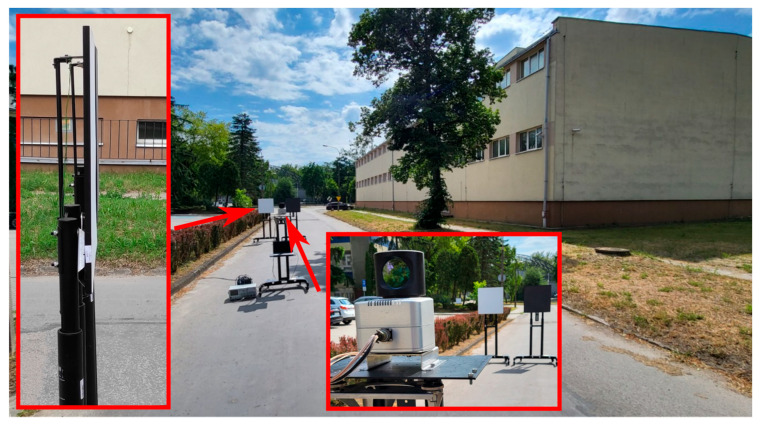
Photo of the field test site showing the scanner and reference targets.

The field tests consisted of performing terrain scans. Two reference targets, “white” (“W”—reflectance 99% at 905 nm, 98.5% at 1550 nm) and “black” (“B”—reflectance 11% at 905 nm, 14% at 1550 nm), were placed in the scanner’s field of view. The targets were positioned at three different distances from the tested scanners (about 10 m, 30 m and 62 m), so that their surfaces were in one plane. The field measurement setup is shown in Figure 8.

**Figure 9:**
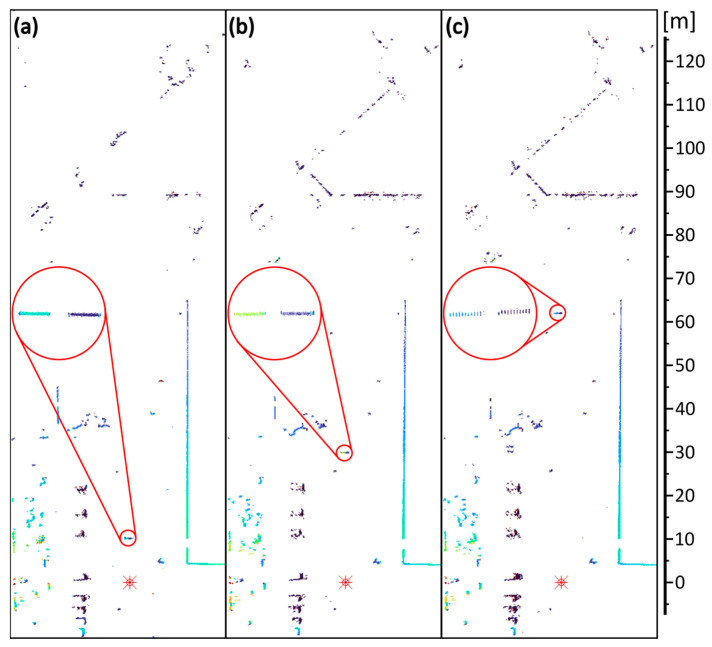
Terrain scans obtained from the SLS scanner: targets at (**a**) 10 m, (**b**) 30 m, and (**c**) 62 m. The reference targets are shown under magnification (red circles).

**Figure 10:**
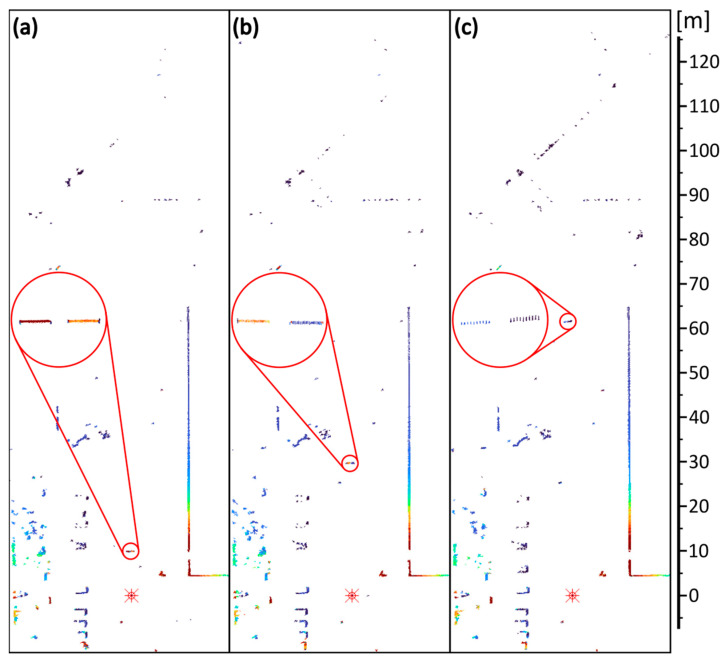
Terrain scans obtained from the FLS scanner: targets at (**a**) 10 m, (**b**) 30 m, and (**c**) 62 m. The reference targets are shown under magnification (red circles).

Figure 9 shows the scans obtained with the SLS, while Figure 10 shows the results from the FLS. In the scans, the reference targets are shown under magnification.

The presented images show that the proposed walk error compensation method used in both scanners works correctly. Reference objects are correctly positioned in the rendered image, regardless of distance and reflectance (signal magnitude). Also, it can be seen that the number of measurement points corresponding to the reference objects decreases with distance, which is related to the finite angular resolution of the scanner.

**Figure 11:**
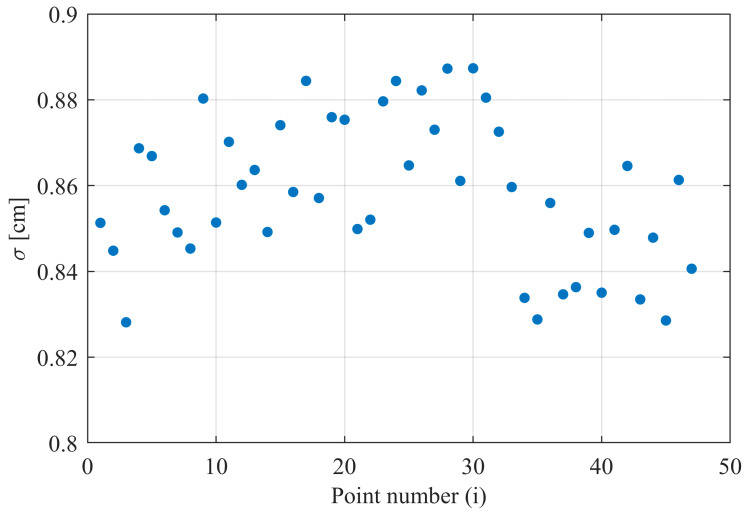
Example graph of the measured distance standard deviation for each point on the target. FLS; target “B”; distance 10 m.

The obtained results were analyzed in terms of distance measurement precision. For this purpose, the standard deviation of the measured distance value was calculated for each point on the reference object, via the application of Equation (5). Figure 11 shows an example of such an evaluation:

(5) $$\bar{\sigma }=\frac{\sum_{i=1}^{n}\sigma_{i}}{n}$$

where *i* is the number of points, *n* is the number of points on the target, and *σ_i_* is the standard deviation for point *i*.

The arithmetic mean of the standard deviation values for measurements of reference objects located at three different distances is listed in Table 1.

Based on the results presented in Table 1, it can be seen that the standard deviation of the distance measurement is larger for a reference object with a smaller reflection coefficient and increases for larger distances. This is mainly caused by the deterioration of the signal-to-noise ratio. The noise level in the detection path is significantly affected by background radiation. Therefore, measurements made during sunny weather generally have a slightly higher error rate. The impact of the SNR on distance measurement precision can be represented by Equation (6) [5]:

(6) $$\sigma_{R}\approx \frac{t_{r}c}{2\cdot SNR}$$

where *t_r_* is the rise time of the laser pulse, *c* is the speed of light, and *SNR* is the signal-to-noise ratio.

## 5. Conclusions

This paper presents a novel method of walk error compensation, which is a fundamental challenge for ToF laser rangefinding precision. The proposed approach is based on a measurement of the absolute magnitude of the echo signal using our authorial ESSMC solution. This enables us to efficiently eliminate the walk error for an extremely-large-magnitude echo signal power, even if it corresponds to detection channel saturation. Based on this method, the effective methodology for calibrating the measurement circuit was also proposed.

In order to verify our approach experimentally, the ESSMC and the developed algorithms were implemented in two laser scanners. The tests were performed both in a lab and in field conditions. Both experiments confirmed the effectiveness of the proposed new method. In laboratory conditions, we managed to reduce the distance measurement error from ±25 cm to ±0.5 cm. It was also pointed out that due to differences in the pulse shape, distortion and accuracy of electronic components, the proposed calibration scheme should be carried out individually for each scanner. The calibrated scanners subjected to field tests also proved the effectiveness of the proposed compensation method. Based on the collected results, it was also confirmed that the precision of distance measurement decreases with the reduction in the echo signal, which is mainly due to the deterioration of the signal-to-noise ratio. It should be underlined, however, that in the measurement range, the standard deviation of the distance measurement did not exceed *σ* = 1.8 cm (far less than the spatial length of the used laser pulse).

## Figures and Tables

**Table 1:** Results of mean standard deviations for measurements to reference targets.

Scanner		SLS	FLS
		$\bar{\sigma }$ (cm)	$\bar{\sigma }$ (cm)
10 m	B	1.477	0.858
	W	1.353	0.833
30 m	B	1.463	1.113
	W	1.393	0.919
62 m	B	1.758	1.700
	W	1.380	0.969

## Data Availability

Data are contained within the article.
